# Impact of super-spreaders on COVID-19: systematic review

**DOI:** 10.1590/1516-3180.2020.0618.R1.10122020

**Published:** 2021-02-15

**Authors:** Ana Paula Schmitz Rambo, Laura Faustino Gonçalves, Ana Inês Gonzáles, Cassiano Ricardo Rech, Karina Mary de Paiva, Patrícia Haas

**Affiliations:** I Undergraduate Student, Speech Therapy, Universidade Federal de Santa Catarina (UFSC), Florianópolis (SC), Brazil.; II Undergraduate Student, Speech Therapy, Universidade Federal de Santa Catarina (UFSC), Florianópolis (SC), Brazil.; III PhD. Professor, Cardiology and Exercise Medicine Group, Physiotherapy Department, Heath and Sport Sciences Center, Universidade do Estado de Santa Catarina (UDESC), Florianópolis (SC), Brazil.; IV PhD. Professor, Physical Education Department, Universidade Federal de Santa Catarina (UFSC), Florianópolis (SC), Brazil.; V PhD. Professor, Speech Therapy Department, Universidade Federal de Santa Catarina (UFSC), Florianópolis (SC), Brazil.; VI PhD. Professor, Speech Therapy Department, Universidade Federal de Santa Catarina (UFSC), Florianópolis (SC), Brazil.

**Keywords:** Coronavirus infections, COVID-19 [supplementary concept], Pandemics, Respiratory diseases, Transmission, New coronavirus disease.

## Abstract

**BACKGROUND:**

Spreader and super-spreader are terms that refer to people who have greater potential for disease transmission, to infect other people.

**OBJECTIVE:**

To present scientific evidence regarding the impact of COVID-19 spreaders.

**DESIGN AND SETTING:**

Systematic review of the literature (using the PRISMA framework), performed at the Federal University of Santa Catarina (UFSC), Florianópolis (SC), Brazil.

**METHODS:**

A search for articles was carried out in the SciELO, LILACS, PubMed, Scopus, Bireme and Web of Science databases. A search for gray literature was also conducted via Google Scholar. There was no restriction regarding place or language, and the search covered the period from January 2010 to August 2020. Studies were selected based on a combination of descriptors from the Medical Subject Headings (MeSH).

**RESULTS:**

Isolated cases of people diagnosed with COVID-19 who were classified as super-spreaders were found. They had been classified thus because they may have had greater potential for infecting other individuals. However, greater numbers of interventions are needed in order to identify and manage COVID-19 cases. There is little evidence regarding this detection, which further hinders recognition and understanding of super-spreading events.

**CONCLUSION:**

The scientific community needs greater depth of evaluation and understanding of how these patients physiologically develop the ability to propagate COVID-19 more intensely. A simpler way of tracking them is also necessary, given that many infected people are asymptomatic. Many patients also have mild symptoms, suggesting that these individuals could also be classified as possible COVID-19 spreaders.

PROSPERO Number: ID 217874 (submitted for publication and is being assessed by the editorial team).

## INTRODUCTION

The coronavirus is a zoonotic virus in the *Coronaviridae* family, a type of viruses that cause respiratory infections, first described in 1965. It is thus named due to its profile, which resembles a crown under a microscope.^1^ People diagnosed with COVID-19 usually develop signs and symptoms that include mild respiratory problems and persistent fever, on average five to six days after infection.^1^ The symptoms normally begin with fever in combination with a dry cough and fatigue, potentially leading to respiratory difficulties.^2^

Spreader and super-spreader are terms that refer to people with a propagation potential greater than the average (i.e. one person propagates to another three), to infect other people.^3^ It is believed that about 10% of the cases of COVID-19 may be responsible for up to 80% of the propagation, which can be attributed to super-spreaders.^3^

The known characteristics of COVID-19 that cause concern in super-spreading events include the number of pneumonia cases relating to COVID-19, the existence of person-to-person transmission, the mean age of 61 years among individuals infected and the possibility that asymptomatic people may be an important source of infection.^4^ Furthermore, the presence of super-spreaders was previously reported during the courses of the severe acute respiratory syndrome (SARS) and Middle East respiratory syndrome (MERS) pandemics.^5^

COVID-19 has been pronounced to be a pandemic global health emergency by the World Health Organization.^6^^,^^7^ It has already killed more than 680,000 people, among the more than 17 million confirmed cases worldwide.^7^ Hence, understanding the physiology of such spreaders and controlling over-propagation is essential, in order to be able to combat the pandemic and, especially, understand the mechanisms through which the numbers of cases in different populations are accelerated.^8^

## OBJECTIVE

The main guiding objective of this study was to ascertain the impact of super-spreaders on COVID-19, so as to answer the following question: What is the impact of super-spreaders on the propagation of COVID-19?

## METHODS

### Protocol

This systematic review was carried out following the Preferred Reporting Items for Systematic Reviews and Meta-Analyses (PRISMA) framework.^9^ Scientific articles were searched by two independent researchers in the MEDLINE (PubMed), LILACS, SciELO, Scopus, Web of Science and BIREME databases, with no language or place restrictions. The search covered the period from January 2010 to August 2020. Additionally, a manual search was conducted on the references of the articles that had been included through the database search, along with a search for gray literature using Google Scholar.

The search was structured and organized based on the PICOS framework - an acronym for target Population, Intervention, Comparison, Outcomes and Study type. The population of interest or health problem (P) corresponds to the patients; intervention (I) concerns the intervention applied; comparison (C) refers to the spreaders; outcome (O) refers to COVID-19; and the study types (S) included in the review encompassed descriptive studies, cross-sectional studies, observational studies, case reports, case-control studies, controlled clinical trials and cohort studies (Table 1).

**Table 1.** Description of the PICOS components

Acronym	Definition
P	Spreaders
I	COVID-19
C	Transmission
O	Disease
S	Cross-sectional studies Observational studies Case reports Case-control studies Controlled clinical trials Cohort studies

### Search strategy

The descriptors were selected based on the Health Sciences Descriptors (DeCS) and Medical Subject Headings (MeSH), given their wide usage by the scientific community to index articles in the PubMed database. After the search for descriptors, the search in the other databases was adjusted. The following combination, with its Boolean operator, was firstly proposed for the search: [(covid 19) AND (spreaders)]. This search was concentrated on August 2020.

#### Eligibility criteria

The study designs included in the search were descriptive studies, cross-sectional studies, observational studies, case reports, case-control studies, controlled clinical trials and cohort studies. They were included with no restriction of language or place, and the search covered articles published from January 2010 to August 2020. The inclusion and exclusion criteria for this search are shown in Table 2.

**Table 2.** Summary of the inclusion and exclusion criteria

Inclusion criteria	
Design	Cross-sectional studies Observational studies Case reports Case-control studies Controlled clinical trials Cohort studies
Place	No restriction
Language	No restriction
Exclusion criteria	
Design	Letters to the editor Guidelines Reviews of the literature Systematic reviews Narrative reviews Meta-analyses
Studies	Unclear studies Poorly described or inadequate studies
Type of publication	Abstract only

#### Risk of bias

The quality of the methodology used in the studies included in the review was independently assessed by the reviewers (APSR and LFG), as recommended by PRISMA.^9^ The assessment gave priority to clearly described information. At this point, the review was blind, masking the authors’ and journals’ names to avoid any potential bias and conflict of interests.

#### Exclusion criteria

Studies published as letters to the editor, guidelines, literature reviews, narrative reviews, systematic reviews, meta-analyses and abstracts were excluded. Studies with unclear, unavailable or no descriptions were also excluded (Table 2).

### Data analysis

The data for the eligibility process were extracted and collated in a spreadsheet designed for systematic reviews that had been developed in the Excel software, version 16.0 (Microsoft, Redmond, WA, United States). This was done by two researchers: the extracted data were initially entered by one of the researchers, and then checked by the other one.

#### Study selection method

Initially, the eligibility reviewers (APSR and LFG) were calibrated by PH, KMP and AIG to conduct the systematic review. After this process and after having any queries answered, the two eligibility reviewers (APSR and LFG) independently examined the titles and abstracts. Articles in which the title was on-topic but for which no abstract was available were also obtained and analyzed in full. Afterwards, the eligible studies were obtained and assessed in full. In specific cases, when a potentially eligible study presented incomplete data, it was envisaged that the authors of that study could be contacted via e-mail for further information. However, in the end, this measure was not necessary. When the reviewers disagreed, a third one (PH or KMP) was involved in the final decision.

#### Data collected

After the screening, the texts of the articles thus selected were reviewed and data were extracted by two authors (APSR and LFG) in a standardized manner under supervision by the other four (KMP, PH, AIG and CRH). The following information was identified: year of publication, place where the study was conducted, language of publication, type of study, sample, method, result and conclusion of the study.

#### Clinical result

The clinical result of interest consisted of ascertaining the impact of super-spreaders on COVID-19. Articles in which this approach was not used were not included in the sample for this review of the literature. The analysis of the present study was particularly limited by the rather small number of published articles addressing this topic that were retrieved. A limited number of patients were included in these articles. The fact that few articles had addressed this topic suggests that it is an innovative one that requires further studies with a larger sample size, in order to ascertain the real significance of super-spreaders.

## RESULTS

Based on the descriptors that had been chosen, the scientific databases were consulted. The results thus obtained are presented in Table 3.

**Table 3.** Classification of the references obtained from the PubMed, SciELO, LILACS, Web of Science and Scopus databases

Descriptors	Number of articles	References excluded	Reason	References selected	Database
[(covid 19) AND (spreaders)]	24	22	Excluded based on the title (15); excluded based on the abstract (4); duplicated (3).	2	PubMed
[(covid 19) AND (spreaders)]	17	15	Excluded based on the title (8); excluded based on the abstract (6); duplicated (1).	2	LILACS
[(covid 19) AND (spreaders)] [(covid 19) AND (espalhadores)]	-	-	-	0	SciELO
[(covid 19) AND (spreaders)]	-	-	-	0	Web of Science
[(covid 19) AND (spreaders)]	-	-	-	0	Bireme
[(covid 19) AND (spreaders)]	-	-	-	0	Scopus
Total	41	37		4	LILACS and PubMed

**Figure 1.**
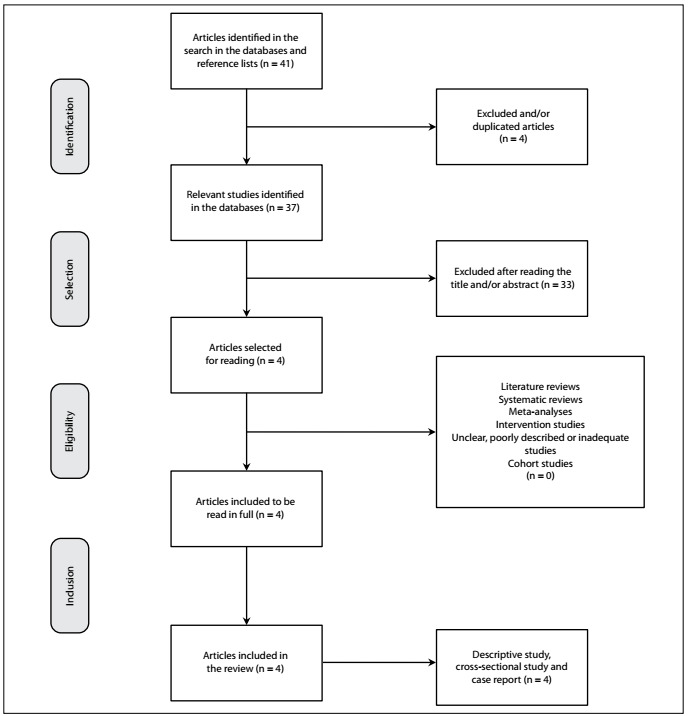
Flowchart of the search for articles and their analysis.

Initially, 41 articles were selected, which decreased to 37 after excluding the repeated ones. The titles and abstracts were then analyzed and 33 papers were excluded because they were not directly related to the topic proposed for investigation here. Hence, four articles were admitted for the final analysis, in which they were evaluated in full by the reviewers. These articles were designed as descriptive studies, cross-sectional studies and case reports (Figure 1).

The main characteristics extracted from each of the articles included in this review are described in Table 4.

**Table 4.** Synthesis of the articles included

Author, year and country	Objective	n	Results	Conclusion
Li et al.,2020, China^10^	To analyze clinical and transmission characteristics of 25 cases of COVID-19 in a thoracic department.	25 people diagnosed with COVID-19.	76% of the cases of COVID-19 were confirmed through a positive COVID-19 nucleic acid test, and 6 cases (24%) were considered suspicious and were diagnosed as presenting clinical manifestations. Greater age and presence of chronic obstructive pulmonary disease (COPD) were significantly associated with the seriousness of the disease and deaths among patients with COVID-19. One possible super-spreader was found, given that 11 people who had been in contact with this person tested positive for COVID-19.	COVID-19 is associated with poor prognosis for patients undergoing thoracic surgery, especially those with COPD. Also, implementing comprehensive protective measures is important for controlling hospital infection.
Zhang et al.,2020, China^11^	To analyze COVID-19 transmission and its underlying causes.	135 inhabitants of the city of Tianjin who had been diagnosed with COVID-19.	135 cases in the city of Tianjin were included in the study. One super-spreader, who infected 6 people in the city, was identified. Based on simulations, the outbreak in Tianjin might have caused 165 infections, had control measures not been implemented in January.	The analysis suggests that heterogeneous COVID-19 transmission may result in a super-spreading event. Further study is necessary in order to verify the heterogeneity of this transmission in other populations and the contributing factors. These matters are crucial for development of measures to contain the pandemic.
Xu et al.,2020, China^12^	To obtain more detailed data involving domestic configurations, in order to have a better understanding of COVID-19 transmission dynamics, based on reconstruction of a database.	643 transmission groups, reconstructed based on 9,120 cases of COVID-19 in China. Out of these, 34 cases were identified as super-spreaders.	34 primary cases were identified as super-spreaders, with 5 super-spreading events within families. The risk of being infected outside the home was greater for people aged 18 to 64 years, whereas the risk of infection at home was greater for younger and older people.	Greater numbers of interventions in COVID-19 transmission are required, given the barriers against identifying and managing the cases, which are due to the non-negligible frequency of super-spreading events.
Lin et al.,2020, China^8^	To understand super-spreading events and the reasons behind the efficiency of their transmission capacity, based on a case report.	A specific case of one super-spreader.	The super-spreader reported in this case infected 28 people, among whom one was asymptomatic. These 28 people infected another 49, among whom 10 cases were asymptomatic.	It can be suggested that a super-spreader may have the following characteristics: 1) having a high viral load; 2) taking longer to eliminate the virus; 3) not necessarily being a seriously ill patient; and 4) being active in social activities and having the chance to be in contact with many people in a short time.

COVID-19 = coronavirus; COPD = chronic obstructive pulmonary disease.

Li et al.^10^ analyzed the clinical and transmission characteristics of 25 cases of COVID-19 in a department of thoracic surgery, in order to better define these characteristics. Out of the 25 cases analyzed, 13 were men and 12 were women; these patients’ mean age was 61 years. No information was obtained on how transmission occurred in that environment. Nevertheless, among the 25 cases observed, one individual proved to be a super-spreader who possibly infected another 11 people.

Zhang et al.^11^ analyzed 135 cases in the city of Tianjin, among which 72 were men and 63 were women. They identified one case of a super-spreader who caused six infections. This analysis revealed heterogeneity in COVID-19 transmission.

Lin et al.^8^ aimed specifically to understand super-spreading events (SSE) and identify the reasons behind the super-spreader’s capacity for transmission. They analyzed a case in which one person infected another 28 people.

Xu et al.^12^ stated that greater interventions would be necessary in order to identify and manage cases of COVID-19, given that the barriers preventing their detection reman large. This also makes it difficult to recognize super-spreading events. They reached this conclusion through an analysis on 643 transmission groups that were reconstructed from 9,120 cases of COVID-19 in China. Among these cases, 34 individuals were identified as super-spreaders.

Li et al.^10^ also reported that it was important that measures to combat COVID-19 should encompass protective measures to avoid hospital infection, given that chronic obstructive pulmonary disease (COPD) has been found to be a risk factor for worsening the condition of people infected with COVID-19, with a likely scenario of poor prognoses and deaths.

Zhang et al.^11^ stated that it was not possible to identify the underlying factors contributing to super-spreading. Studies going into greater detail would be required, considering that identifying these contributory factors was of immense importance in developing measures aimed at controlling the pandemic.

Lin et al.^8^ reported with regard to super-spreaders that, although their case was an isolated one, they had managed to identify some general characteristics that are probably present among super-spreaders. These characteristics would include the following: 1) having a high viral load; 2) taking longer to eliminate the virus; 3) not necessarily having a serious condition; and 4) probably being active in social events, with greater chances of being in contact with many people within a short time.

## DISCUSSION

Studies and research on the subject of super-spreading are still scarce due to the difficulty in isolating such findings. This review made it possible to see that there are various perceptions of the characteristics of a super-spreader. Some authors have stated that a super-spreader can be any person with a contamination power greater than the average (contaminating three people),^11^ whereas others have stated that an individual can only be considered to be a super-spreader when he or she infects more than 10 other people.^12^

Several issues relating to the form of propagation can be highlighted. Firstly, this needs to be in contact with many people, which suggests that participation in a social event is required in order to give power to these contacts. Moreover, it can be supposed that this person has a high viral load, while not necessarily having a serious condition because of their COVID-19 contamination, and will take longer to eliminate the virus.

Various cases of asymptomatic contaminated individuals who are less likely to transmit the virus have already been reported. However, it is still difficult to establish a process through which the contamination can be identified and controlled.^3^ There have also been reports of people whose first test came out negative but who were diagnosed with COVID-19 in the retest.^3^

Cave^3^ considered that use of the term super-spreader was problematic because it could put blame on the people who were supposedly causing this greater propagation, thus hindering identification of cases for scientific analysis. Furthermore, the term may have different meanings in given contexts. In the initial stage of a pandemic, it is mainly focused on people who have contact with many others, as stated in one of the studies examined in this analysis (Lin et al.^8^). At other stages of the pandemic, this may not make so much sense (for instance, in a situation of social isolation and distancing).

Early diagnosing of COVID-19 is essential for controlling its dissemination. Countries like New Zealand managed to control the pandemic, through eliminating community transmission with strategies such as large-scale testing to track and quickly detect cases and implementation of lockdowns, border controls and actions to promote health education. China also rapidly implemented measure for detection of COVID-19, which involved isolated of cases and tracking of all individuals with whom the cases had been in contact with, while always providing good clinical care for infected people.^13^

With regard to COVID-19 treatment, there is still no evidence to prove the effectiveness of some of the medications that have been considered for such treatments, such as hydroxychloroquine and azithromycin. According to Vieira et al.,^14^ it has been hypothesized that these medications can change the course of the disease, through decreasing morbidity and more quickly diminishing the viral load. However, because there is no scientific proof, physicians should only use these treatments if patients explicitly agree to this, by signing an informed consent form.

Jayawardena et al.^15^ published a clinical trial on viral diseases in which they sought to identify nutritional factors that might influence treatment and control of COVID-19. They identified potential benefits from use of vitamins (A and D), especially in populations that lacked them. In addition to vitamin supplementation, use of trace elements (selenium and zinc) was found to be effective for immunomodulation against respiratory viral infections. Some nutraceuticals and probiotics may also have a role in increased immunological functions, and micronutrients have been shown to be beneficial for older adults with nutritional deficiency. Hence, vitamins, trace elements, nutraceuticals and probiotics have been identified as beneficial for combating viral infections. They may therefore be useful in the effort to prevent and manage COVID-19.

On the other hand, Bomfim and Gonçalves^16^ evaluated food supplements for controlling aggravated COVID-19 infection or its spread. They concluded that there was no scientific evidence to show that substances could have a role in controlling this disease, given that only some food supplements proved to be effective (and only rather weakly) in treating specific symptoms of this disease.

## CONCLUSION

The results from these studies suggest that there is some difficulty in detecting COVID-19 super-spreaders, considering that many infected people are asymptomatic. Because of this great likelihood that the symptoms of COVID-19 will only be mild, the disease tends to be itself super-spreading. Another factor that also hinders detection of super-spreader individuals is that the term super-spreader is often incorrectly used. People who are in contact with larger numbers of other people are inevitably more likely to infect more individuals than are those who have contact with few or no people. However, this does not mean that someone who has infected more people has the biological characteristics that demonstrate greater potential to contaminate other people - which is what would identify this individual as a super-spreader.
